# Confronting Lexical Choice and Error Distribution in Written French: New Insights into the Linguistic Insecurity of Students with Dyslexia

**DOI:** 10.3390/brainsci11070922

**Published:** 2021-07-12

**Authors:** Audrey Mazur-Palandre, Matthieu Quignard, Agnès Witko

**Affiliations:** 1 Laboratoire d’Excellence ASLAN & Laboratoire CNRS ICAR, University of Lyon, 69007 Lyon, France; 2 Laboratoire CNRS ICAR, 69007 Lyon, France; matthieu.quignard@ens-lyon.fr; 3 Laboratoire CNRS DDL, 69007 Lyon, France; agnes.witko@univ-lyon1.fr; 4 Institut des Sciences et Techniques de Réadaptation, Université Lyon 1, 69008 Lyon, France

**Keywords:** dyslexia, higher education, written production, spelling

## Abstract

The main goal of this paper is to analyze written texts produced by monolingual French university students, with and without dyslexia. More specifically, we were interested in the linguistic characteristics of the words used during a written production and of the type of word errors. Previous studies showed that students with dyslexia have difficulties in written production, whether in terms of the number of spelling errors, some syntactic aspects, identification of errors, confusion of monosyllabic words, omissions of words in sentences, or utilization of unexpected or inappropriate vocabulary. For this present study, students with dyslexia and control students were asked to produce written and spoken narrative and expository texts. The written texts (N = 86) were collected using Eye and Pen© software with digitizing tablets. Results reveal that students with dyslexia do not censor themselves as regards the choice of words in their written productions. They use the same types of words as the control students. Nevertheless, they make many more errors than the control students on all types of words, regardless of their linguistic characteristics (length, frequency, grammatical classes, etc.). Finally, these quantitative analyses help to target a rather unexpected subset of errors: short words, and in particular determiners and prepositions.

## 1. Introduction

### 1.1. Dyslexia in Higher Education in France

Higher education institutions are welcoming an increasing number of students with disabilities, in the United Kingdom [1,2], for instance, or in France [3]. In France, for the 2018–2019 academic year, 34,553 students enrolled in higher education are reported as having a disability, which represents 1.64% of students and an increase of 15.2% from the previous academic year. The Ministry of Higher Education, Research and Innovation mention that this represents a 12.4% increase in students with disabilities per year, since 2005 [3]. Among them, 6949 are declared to have speech and language impairments, which represents 20.1% of students with disabilities. This number may hide a much more concerning reality. Indeed, in 2015 and 2016, two surveys on dyslexia in higher education were conducted at the University of Lyon. The objective was to understand the needs and difficulties of students with dyslexia at the University of Lyon. Various questions about different topics: ranging from student life to learning and diagnosis were asked. One of the questions was “are you declared to the disability service of your institution?”. For the first questionnaire (2015), two groups of students were selected among the participants, 97 students with dyslexia and 97 control students (matched for gender, age, grade level). Statistical tests were performed on each of the questions to see what difficulties common difficulties to the majority of students and which difficulties are more specific to students with dyslexia (for a presentation of several results, see [4]. Among the 97 students with dyslexia, only 42 responded that they were declared to their institution’s disability service. In the second questionnaire (2016), among the 83 students with dyslexia selected for statistical analyses of questionnaire responses, only 38 responded that they were declared to their institution’s disability service (for a presentation of several results, cf. [5]). These two results reveal that, potentially, one out of two students with dyslexia is not declared to their institution’s disability service. Thus, the number of students with dyslexia in higher education may be higher than reported.

The classification used by Kabla-Langlois and Weisenburger [3] does not allow to know the number of students according to the disorder type (dyslexia, dysorthographia, dyscalculia, dyspraxia, dysgraphia, or dysphasia) out of the 6949 with language and speech disorders. Nonetheless, calculations using enrollment numbers by the institution can give a representation of a possible distribution. For example, at the Catholic University of Lyon, in 2019–2020, out of the 8000 students enrolled, 208 students are declared to the institution’s disability mission, 89 of whom had dyslexia (thus 42% of students with disabilities) [6].

These indicators show that the number of students with dyslexia increases and that it is becoming urgent to have a better understanding of dyslexia, its manifestations, and resulting difficulties. There is a growing need for higher education institutions to understand this disorder in order to bring responses to needs and difficulties. The present study is part of this objective by focusing on the textual production of students with dyslexia. The productions of 21 students with dyslexia and 22 non-dyslexic students matched in gender, age, and school level were analyzed. Specifically, we examined the words used by dyslexic and non-dyslexic students: do students with dyslexia use words with specific linguistic features (number of letters, number of syllables, frequency, orthographic consistency, etc.) that differ from those of control students. In addition, we performed the same analyses on word errors to see if students with dyslexia made grammatical and spelling errors on specific words. These analyses provide information about the difficulties of students with dyslexia in written production and complete literature about dyslexia in adulthood. Indeed, although there are important published data on children with dyslexia, it seems that very little addresses this disorder in adulthood. Moreover, most of the studies are from a psychological or neuropsychological perspective and few from a psycholinguistic one, implying a focus on behavioral aspects: the direct impact of the disorder on natural written production. In addition, despite the increase of students with dyslexia in higher French education institutions and its negative impacts on education, there remains a paucity of studies in France, on French students, from a psycholinguistic perspective. To finish, our results will guide remediation, giving some keys of understanding et precisions about spelling difficulties.

### 1.2. What is Dyslexia?

#### 1.2.1. Normative Definition

Dyslexia is classified as a specific learning disorder, which is classified as a neurodevelopmental disorder, in the DSM 5 [7]. This category of specific learning disorders groups together all signs pertaining to reading, writing, or calculation disorders, under common diagnostic criteria. Thus, they involve difficulties—with skills significantly below those expected for age and significantly interfering with academic performance—in learning and using academic skills for at least six months, with at least one of the following symptoms: inaccurate, slow, or labored word reading; difficulty understanding the meaning of what is read; difficulty with spelling; difficulty with written expression; difficulty mastering number sense, number facts, or computation; and difficulty with mathematical reasoning. Specific learning disabilities involve skills that are significantly below those expected for a given chronological age, negatively and significantly interfering with academic performance at school or university. These developmental hindrances appearing during the schooling years may manifest themselves only when demands exceed the individual’s limited abilities, and cannot be explained by other impairments (intellectual, hearing, visual, etc.). The most common manifestation of specific learning disabilities is dyslexia, which refers to a learning profile, characterized by difficulties in recognizing common words accurately or fluently and poor decoding and spelling skills. It is estimated at a worldwide prevalence of 10% [8] (and in France between 6 and 8% [9,10,11]). These difficulties can then lead to a reduced experience in reading which could impede the growth of the child’s vocabulary and general knowledge [12]. In the classroom, these difficulties, for example, are often unexpected in relation to the individual’s other cognitive abilities and the instruction provided in the classroom. It may be surprising to see the difference in the oral and written skills of the same child on the same topic. Problems with reading comprehension may be one of the indirect consequences of these reading difficulties.

This normative definition of dyslexia can be complemented by specific criteria [13]: a deficit in phonological processing is noted [14]; dyslexia is lifelong; there are exclusionary factors to differentiate a person with dyslexia from a person with a learning disability (absence of visual, attentional, mental, emotional, etc.); there is adequate access and exposure to learning [15]. Furthermore, dyslexia cannot be attributed to a lack of motivation to learning to read [16]. In conclusion, dyslexia is a complex and multifactorial disorder due to the fact that a single biological cause can lead to multiple cognitive deficits [17].

#### 1.2.2. An Evolving Definition

Faced with this normative positioning of dyslexia positioned within a nosographic classification of specific learning disorders, a transdisciplinary vision drives us to rethink the defining perimeter of dyslexia [18]. It also seems today, essential to take into consideration the “evolutionary trajectory” [19] of each individual. Indeed, a disorder undergoes changes throughout a person’s life and taking into account individual trajectories allows for the redefinition of the contours of the disorder, and its impact in different areas of life [20]. Speech therapy practice testifies that dyslexia is a disorder that can evolve favorably due to treatments or compensations implemented by a person with dyslexia, with the limit of spelling difficulties that most adults with dyslexia complain about [21,22]. Indeed, the scientific literature considers dysorthographia as a disorder consubstantial with dyslexia [12,23]. Nor is dyslexia limited to a reading and spelling problem [24,25]; it involves difficulties related to textual production, impacting syntax, lexicon, high-level processes, etc. [24,26,27,28].

### 1.3. Written Production and Dyslexia

#### 1.3.1. Written Production: A Complex Task

Written production is a difficult task implying several types of cognitive processes. Among them, there are the phoneme/grapheme conversion system and the grapho-motor processes, which are automated in children with typical development, approximately between 9 and 12 [29]. From this period, children mobilize cognitive resources, not only for the management of the conversion system but also for high-level writing processes, such as text planning or revision [30]. Around 12–15 years of age, children can manage more easily all the processes deployed during a writing task [30], but it is not until 16 years of age that they can totally manage the planning processes [31].

One of the essential steps in becoming an expert script-writer is managing the spelling codes, which “differ greatly in the consistency of their mappings between the minimal units of the written language (graphemes) and the minimal units of the spoken language (phonemes)” [32], p. 4. Grapheme/phoneme and phoneme/grapheme inconsistencies come from the fact that a grapheme can have several pronunciations and a phoneme can correspond to several graphemes [32]. According to the language, this conversion grapheme/phoneme and phoneme/grapheme can be more or less consistent. A cross-linguistic study showed large variations in seven orthographies [33]. For instance, certain systems are more consistent, such as in Finnish with a transparent conversion system, or less consistent, such as in English with an opaque system. French occupies an intermediate position, as it is a relatively opaque language in which phoneme/grapheme relationships are more unstable than grapheme/phoneme relationships [34,35]. Many studies showed the impact of the consistency of grapheme/phoneme and phoneme/grapheme on children spelling acquisition in English [36,37,38] and in French [39,40]. Indeed, spelling acquisition is faster and simpler with transparent orthographies, implying a strong consistency rather than with opaque orthographies, implying a weak consistency. Furthermore, the high cost of the spelling dimension is said to result in “poor compositional performance” [41], p. 397.

The information processing system for writing is therefore a system with a limited capacity [42]: if the cognitive resources allocated to transcription remain significant, fewer resources will be available for the other processes required, such as planning or text editing [41,42]. What happens then for adults who cannot fully automate the phoneme/grapheme and grapheme/phoneme system conversion, such as students with dyslexia? What is the impact on their written texts and on the processes requiring considerable cognitive resources, such as revision processes?

#### 1.3.2. Spelling and Dyslexia in Adulthood

Studies show that adults with dyslexia continue to experience difficulties with writing [2,4,5,27,28,43,44,45,46,47,48,49,50,51,52] in spite of remediation programs that reduce the difficulties but do not eliminate the anomalies [52]. Many students with dyslexia have problems with composition organization, handwriting, punctuation, and redrafting [53]. Indeed, students with dyslexia, during a proofreading task, are less successful and efficient than non-dyslexic students [2,26,49]. Horowitz and Breznitz [46] suggest that people with dyslexia have a deficit in the error detection mechanism so that they cannot identify all spelling errors and then correct them. Moreover, several studies about English students with dyslexia reveal that their written texts have still more errors than control students, even if they proofread their texts [54,55]. In a report on dyslexia in higher education [53], p.28, it is mentioned that students with dyslexia encounter a variety of difficulties when writing a text, including: “an intractable spelling problem, often concealed by the use of an automatic spellchecker; confusion of small words such as which / with; omission of words, especially when the writer is under pressure; awkward handwriting and/or slow writing speed”. Moreover, an unexpected difference is observed between oral and written expression, with better quality in oral productions than in the written productions, in terms of structure, self-expression, and relevance of the vocabulary used [53].

Other studies complete these results. Students with dyslexia realize more spelling, syntactic [2,26,28,43,47,48,51] and morphosyntactic errors [1,56,57] than students without dyslexia. Regarding spelling errors, students with dyslexia seem to make up to five times the number of errors (semiographic—lexical and morphosyntactic spelling, logographic and morphographic errors—and phonological errors) in their texts than the control students [1,47,56,57]. A qualitative study of grammatical errors reveals that French students with dyslexia make mistakes that control students never do; for instance, errors concerning the number agreement of the verb, some students with dyslexia may make errors of the type: using the plural form for the singular (“cette discordance ne peuvent être toléré “) or using the plural of the nominal phrase for a verbal form by putting “s” instead of “ent” (“les personnes proviennes”) (See, Mazur-Palandre, 2018, for more examples). Concerning morphosyntactic errors, studies about English students with dyslexia show that there are very common types of errors in students with dyslexia texts: pronoun omission/confusion, determiner omission/confusion, preposition omission/confusion [1,56,57]. Moreover, they can use unexpected or inappropriate vocabulary [1,50,58]. Students with dyslexia are described as poorer spellers than students without dyslexia “because of the extra effort and time demanded by spelling” [50], p.11. Several explanations explaining spelling difficulties of students with dyslexia are given [50,58]: first, they still have a phonological impairment that has an impact on their use of phonemic transcription; second, students with dyslexia have problems choosing the correct spelling alternative for one word over another; and thirdly, they retrieved the wrong spelling of a word from orthographic memory.

Moreover, concerning lexical choices, some studies also report that English students with dyslexia produce fewer polysyllabic words than control students [1,26,50] and that they use a “restricted/inappropriate vocabulary” [26], p.26, than control students [1,50,58]. In addition, it is reported that students with dyslexia report avoiding the use of words for which they are uncertain of the spelling, implying a preference for simpler, less sophisticated vocabulary and for words involving simpler spelling [1,26]. However, Hatcher’s [28] work qualifies these conclusions by mentioning that there are no differences in vocabulary level between students with dyslexia and non-dyslexic English students.

So, what can we expect with French students with dyslexia? We can imagine that in written production, they would prefer to use words for which they control the spelling, shorter words, more frequent words, or words with a regular orthographic consistency, which could help them to avoid errors. There is increasing concern about adults and dyslexia and the impact of this disorder on people’s lives, and more specifically on their relationship and performance in writing. Nevertheless, as mentioned before, there is currently little published data about students with dyslexia. With this research, we seek to obtain data and analyses which will help to address this research gap. The aim of this paper is also triple: first, we want to know if students with dyslexia use specific words with specific linguistic characteristics, compared to non-dyslexic students; second, we want to see if students with dyslexia make more errors on all types of words and third, to see if, more specifically, there are any word properties to which students with dyslexia, compared to non-dyslexic students seem to be more sensitive to.

### 1.4. Hypotheses

According to the previous studies and being aware of their problem with the written words and more specifically with spelling, we thus assume that students with dyslexia, who experience orthographic insecurity:

Hypothesis**** **1.** *Use fewer complex words than control students, thus showing a form of self-censorship/inhibition related to linguistic insecurity; they tend to use words with specific linguistic characteristics, which would differ from the words called by non-dyslexic students, such as shorter words, words with regular spelling or frequent words.*

Hypothesis**** **2.** *Make more errors than control students, and this is true for all types of words; for instance, they make more errors on all the words, no matter how long they are or no matter their frequency.*

Hypothesis**** **3.** *Are more sensitive to certain linguistic properties and therefore make more errors on certain types of words, compared to control students; for instance, we can presume that they are more sensitive to the length, and also make more errors on long words than short words. Moreover, we expect that students with dyslexia realize more errors on words with a low frequency and on words morphological inflected, such as nouns or verbs.*

## 2. Methods

### 2.1. Participants

Data collection was carried out in the framework of projects concerning difficulties and needs of French students with dyslexia in higher education (ETUDYS, DYS’R’ABLE and FLEXIDYS projects (Projects co-founded by PEPS CNRS program, the LabEx ASLAN, the Ecole Normale Supérieure de Lyon and the CNRS Laboratory ICAR (CNRS, Université Lyon 2and Ecole Normale Supérieure de Lyon))). There were several steps in these projects: (1) two online questionnaires about difficulties and needs of students with dyslexia at university filled in by 1454 students for the first one (analyses ran on the responses of 97 students with dyslexia and 97 control students matched in gender, age and level of study) and 1472 for the second (analyses ran on the responses of 83 students with dyslexia and 83 control students matched in gender, age and level of study); (2) a speech, language and neuropsychological assessment (N = 30 students with dyslexia and 30 control students) (Written language processing: ECLA 16+ battery [59] and “Le vol du PC” [60]; decoding: reading of isolated words (regular, irregular and pseudo-words), and texts (Le vol du PC and L’Alouette); spelling: dictation of isolated words (regular, irregular and pseudo-words) and text (ECLA 16+), reading comprehension: Le vol du PC text subtests; meta-phonological skills; neuropsychological tests: TAP-M [61]; visuo-attentional skills: Report Global test [62,63](see EVADYS [64]), SIGL test [65], visual search test (n cancellation test, ECLA 16+), and visual and auditory orientation tests (Visioner and Audioner [66]); perceptual reasoning (Matrices), short-term memory and auditory-verbal working memory (Number Memory): tests from the Wechsler scales assessed. Results from this part of the protocol are reported in previous work [4,67]); (3) psycholinguistic task consisting of producing four texts types (spoken narrative, written narrative, spoken expository, written expository). The present paper focuses on the written psycholinguistic data of 21 students with dyslexia and 22 control students (Table 1).

Students with dyslexia diagnosed in childhood all had associated dysorthographia and had received speech and language therapy during their childhood/adolescence. At the time of data collection, only two students with dyslexia out of the 21 participants said that they were registered with the Handicap Service of their institution and thus had additional time to complete exams (none used specific digital tools or were following a remediation program at the time of collection). The students were all monolingual native French-speakers and gave written consent to participate in the assessment and the psycholinguistic task for which they received financial compensation. The exclusion criteria, verified during personal interviews (at the time of the assessment), excluded individuals with hearing or visual deficits or other disorders.

### 2.2. Psycholinguistic Task: Data’s Collection

#### 2.2.1. Protocol: Textual Elicitation

After having filled in the questionnaire and passed speech and neuropsychological assessments, the students carried out the psycholinguistic task. They were asked to produce four texts on the theme of conflict between people, which corresponded to four experimental conditions: oral narrative, written narrative, oral expository, and written expository, following Berman and Verhoeven [68] text production tasks. For the expository text, students were asked to produce a text on problems between people, discussing the theme by presenting their ideas as if in a school presentation. For the narrative text, students were asked to tell a personal story about a conflict they might have experienced. Data collection was conducted during two sessions with a one week-long interval between them in order to avoid the effects of fatigue. Each subject had two appointments. During the first week, the project was presented rapidly to the participants who then watched a video. It was a three-minute video film without words, depicting a variety of short scenes of conflict between people in a school environment. This video was specially made for the Spencer Project (responsible: Ruth Berman). Next, they had to produce a narrative or an expository text in written and spoken modalities. Between the production of the written and spoken texts and in order to avoid transfer (word by word from one text to another), participants were asked to answer a questionnaire, about written and spoken language (reading habits, relationship to the written word, etc.), given orally by the experimenters. During the second week, participants had to produce in written and spoken modalities either a narrative or an expository text. Between the two texts, they also had to answer another questionnaire, about written and spoken language too. Students were therefore divided into two test orders: half produced a written then an oral text and the other half produced an oral then a written text. The text production order was counter-balanced: half of the participants produced an expository text first and then a narrative text and the other half did the opposite. Students had no time limit and were allowed to take all the time they needed to write their texts and to proofread them if they wanted. This present study focuses on written texts. 

#### 2.2.2. Written Data Exploitation

Written data were collected using digitizing tablets via the Eye and Pen© software [69] and transcribed according to CHILDES (https://childes.talkbank.org/ (9 July 2021)) conventions with Transcriber for oral data and in Eye and Pen© for handwritten data and exported into the CLAN software. The productions were divided into clauses—the clause being defined as a unit of meaning made up of a finite or non-finite verb and arguments—and terminal units (TU)—a unit made up of a main clause and all its dependent clauses such as its subordinates. These two units have been shown to be appropriate for the evaluation of syntactic development [70,71]. For this study, our written text corpus consists of 86 text productions (43 expository and 43 narrative), which represent 2328 clauses (expository: 1089; narratives: 1239) and 1126 UT (expository: 515; narratives: 611). Table 2 shows the length of the texts according to text type and group.

ANOVA analyses show that differences in length between students with dyslexia and control students in number of words (F_(1.39)_ = 0.089, *p* = 0.767), clauses (F_(1.39)_ = 1.842, *p* = 0.183), Units T (F_(1.39)_ = 2.501, *p* = 0.122) and clauses per Unit T (F_(1.39)_ = 0.773, *p* = 0.385) were not significant. Moreover, analyses reveal that differences in time duration (duration of written production) between the two groups are not significant (F = 2.07; *p* = 0.164 > 0.1).

For this study, we did not use the functions of CLAN, either for coding or for commands, as we do for other analyses. But, we extracted, from these CLAN transcriptions, all the words and spelling errors, which constituted the two corpora that we analyze in the present paper: (1) the first corpus of all the words used by students with dyslexia and control students (Corpus Words, 16,707 words) and (2) the second corpus of all the words containing an error (Corpus Word Error, 1177 words with errors). Our category of error includes semiographic and phonographic errors, according to the distinction proposed by [72]. Drawing on previous work [73,74,75], among the semiographic errors, we distinguished: (1) lexical morphogrammic errors (non-respect of lexical morphograms: “singulièremant” instead of “singulièrement”, “parends” instead of “parents”); (2) errors concerning non-functional graphemes, double consonants, and final letters (“longtemp” instead of “longtemps”); (3) errors of grammar (number, gender, verbal endings like: “des guerre” instead of “des guerres”, “une personne caractériel” instead of “une personne caractérielle”, “j’ait pu” instead of “j’ai pu”); (4) errors with a logogrammatic dominance (homophones); (5) errors related to ideograms (hyphens, capital letters, apostrophes added or forgotten); (6) segmentation errors (“par ce que” instead of “parce que”). Among the phonographic errors, the following have been distinguished: (1) phonetic errors (omissions, confusions, inversions, and additions of graphemes); (2) phonogrammic errors, altering the phonic value of the word (“saussise” instead of “saucisse”) or not (“diffissile” instead of “difficile”). Moreover, we include words omissions.

Table 3 gives the number of words and words containing an error according to text type and group.

#### 2.2.3. Coding

The purpose of this study is to ascertain: (1) if students with dyslexia use words with specific linguistic features that are different from control students, and (2) if errors of students with dyslexia are made on words with specific linguistic features. To this aim, we coded all words (Corpus Words) with several types of linguistic information annotated on them. For this paper (we had more coding, such as number of letters, graphemes and phonemes, and syllables. However, to avoid some redundancy and because the statistical analyses show the same results for numbers of syllables and length, we decided not to present these measures), for each word were indicated the following features:

- Length properties: numbers of written syllables and word length: short words: 1 to 4 letters; medium words: 5 to 7 letters; long words: more than 8 letters; The three indicators of word length (number of letters, phonemes, and syllables) are all provided by Lexique and Manulex databases and conventionally used in psycholinguistic studies on written or spoken language [32,39].
- Graphemic and phonetic properties: the phoneme/grapheme consistency indicator, the grapheme/phoneme consistency indicator, based on the calculation of Lété, Sprenger-Charolles & Colé [76]; and the phonological structure (consonant/vowel succession);
- Frequency properties: frequency of lemmas (the lemma of a word is actually the form that is used as an entry in dictionaries. In inflected languages like French, these are singular masculine forms for nouns and adjectives and infinitive forms for verbs), according to the Lexique.org lexical database (based on French movie subtitles, number of occurrences per million);
- Syntactic properties: the grammatical class (abbreviation, adjective, adverb, conjunction, determiner, noun, preposition, pronoun or verb); we have also added a category for punctuation;

For instance, the word *tension* (tension), was coded with the following information:

- Length properties: 2 syllables; length: medium word;
- Frequency properties: 2,128,000;
- Graphemic and phonetic properties: the phoneme/grapheme consistency indicator: 71,216, the grapheme/phoneme consistency indicator: 75,92 and the phonological structure: CVCVV;
- Syntactic properties: the grammatical class: noun.

#### 2.2.4. Processing Chain

In order to obtain this linguistic information, a processing chain consisting of several steps was developed. The first step consisted in obtaining the subjects’ textual productions from the transcripts annotated in CLAN software (according to the CHILDES conventions). We used two format conversion scripts (CLAN to XML/TEI, then XML/TEI to raw text) to filter out any pre-existing CLAN annotations (for instance, transcripts containing errors or syntax coding, clause segmentation, etc.) and to replace incorrect words with the targeted correct form. The 86 written texts without any CLAN codes were fully checked by two reviewers to ensure that no words were lost or added in this process.

The second step consisted of tokenizing the corpus (i.e., segmenting it into words according to a language model), using the TreeTagger software and the basic model of French (french-utf8.par). The 67 words of the texts that were not included in the model were added manually so that all 86 written texts could be analyzed. The additions were, for the most part, proper nouns and very specific words, such as *transgender* or *powerpoint*. This process yields, for each word, its lemma and its grammatical category, according to the TreeTagger model (32 categories). At the end of this step, we extracted the complete lexicon of the corpus, which includes 3031 different forms and 16,702 forms in total.

During the third step, we added lexical information in addition to lemma and part-of-speech (POS) produced by TreeTagger. Lexical information was provided by two lexical databases for French: Lexique.org and Manulex-infra (hereafter called Lexique and Manulex). Lexique contains a large set of information about phonetization and word usage frequency (both as to form and as lemma) in French literary texts of subtitled films. Manulex has been developed by Lété, Sprenger-Charolles & Colé [76] for the description of infralexical characteristics of forms (45,000 entries; see [77,78]. This database provides for each word some indicators of orthographic difficulty (or orthographic consistency), computed for the beginning, the middle, the end, and for the whole word. As in the case of TreeTagger, we had to complete by hand the missing information corresponding to the words of our corpus, unknown for those databases. It should be noted that French language contains a large number of ambiguous forms (e.g., “le” may be both a determiner and a pronoun). To solve these ambiguities, and assign the correct information to the targeted form, we based our matching on the form, the lemma, and the POS given by TreeTagger. Since those POS categories do not strictly coincide with those of Lexique and Manulex, we had to reconfigure all POS categories to establish a shared tagset between the three databases. The largest part of the manual enrichment consisted in the computation of orthographic consistency since Manulex has a rather small lexical coverage (it has been developed from French primary textbooks). This manual task concerned 500 forms. For those forms, we are unable to provide the frequency information: this will be left as 0 (which actually means unknown).

On each pair of words in the Corpus Word Error—the word containing an error and its corresponding “correct” target word—we project the lexical information attributed to the target word in the corpus Words. By this transfer of information, we may observe on each error what syntactic function was concerned, or the extent to which the target word was, for instance, length, frequency, graphemic and phonetic properties, and finally syntactic properties. 

## 3. Results

### 3.1. Preliminary Information

Three analyses were performed. First, all the words from the texts of the students with dyslexia (Dys.) and the control students (Control) texts (Corpus Words, 16,707 words) were extracted and included in a database in which for each word, several types of linguistic information were given (cf. 4.2.3). Analyses were run on the measures presented in the previous section and for each of them, analysis of variance (comparison of means, inter ANOVA) were performed with an inter-individual factor, Group (students with dyslexia and control students). The threshold of significance retained is *p* < 0.05 and the standard deviation is indicated (S.D.).

Secondly, we performed statistical analysis on the errors of students with dyslexia and control students’ errors. For each of the categories presented above, the average number of errors per category according to the number of words in the text (proportion) was calculated, according to the Group factor. These categories included qualitative variables (syntactic categories, and word length—short, medium, and long) and quantitative variables (number of errors, syllables, grapheme/phoneme, and phoneme/grapheme consistency indicators, phonological structure indicator, and frequency). For the qualitative variables, analyses of variance (comparison of means, ANOVA) were performed with an inter-individual factor, Group (students with dyslexia and controls students). The threshold of significance retained was *p* < 0.05. For quantitative variables, multivariate analyses of variance (MANOVA) with an inter-individual factor, Group (students with dyslexia and control students). The threshold of significance retained is *p* < 0.05. When errors distributions with quantitative variables are to be compared (e.g., frequency), one cannot proceed with proportions anymore. One has to discretize the continuum of values into a limited set of ranges and then count and compute proportions of errors falling within each range. Some variables are already discrete, with a small number of values (for example, the syllable length or the phonological structure): we exploit those values as if they were categorical variables. Otherwise, when there are too many different discrete values (number of letters, of graphemes) or when variables are continuous (frequency, consistency), we rely on the distribution quartiles, computed on the texts produced by control students. Those quartiles consist of four sets of range whose boundaries are calculated to divide the distribution of control students into four sets with each the same number of errors (25%). We assume, using a MANOVA, that the distribution of students with dyslexia, using the same boundaries as control students, will show a different number of errors in at least one quartile. For each quantitative variable, we produce the values defining the quartiles (Q1, Q2, Q3, and Q4), the proportions of errors measured in each quartile and each group, and eventually the result of the MANOVA.

Thirdly, all the errors observed in students with dyslexia and control texts were extracted and we built a database in which, for each error, several types of linguistic information were given about words containing the error (cf. 4.2.3). As for the corpus of words, analyses were run on the different measures quoted above (length, graphemic and phonological properties, frequency, and syntactic properties). For each of these measures, analyses of variance (comparison of means, ANOVA) were performed with an inter-individual factor, Group (students with dyslexia and control students). The threshold of significance retained is *p* < 0.05.

### 3.2. Words Characteristics

#### 3.2.1. Length Indicators

Mean comparison tests (ANOVAs) were performed on the number of syllables per words (F_(1,41)_ = 0.024; *p* > 0.5) and on the proportion of short (F_(1,41)_ = 0.014; *p* > 0.5), medium (F_(1,41)_ = 0.186; *p* > 0.5) and long words (F_(1,41)_ = 0.081; *p* > 0.5), according to the group factor (students with dyslexia; control students). The mean differences between the two groups for these three measures are not significant. Indeed, analyses show that students with dyslexia use words with a similar length to the control students, both in number of syllables (Dys: number = 1.5 (S.D. = 0.1); Control: number = 1.5 (S.D. = 0.1)) and number of characters. Students with dyslexia and control have a proportion of 0.64 short words (1–4 letters) (Dys. S.D. = 0.05; Control S.D. = 0.04), a proportion of 0.21 medium words (5–7 letters) (Dys. S.D. = 0.04; Control S.D. = 0.04), and a proportion of 0.15 long words (8 letters and more) (Dys. S.D. = 0.05; Control S.D. = 0.05).

#### 3.2.2. Graphemic and Phonetic Properties

Mean comparison tests (ANOVA) were performed on the spelling consistency indicators (grapheme-phoneme consistency (F_(1,41)_ = 0.394; *p* > 0.5); phoneme-grapheme consistency (F_(1,41)_ = 0.523; *p* > 0.1) and on the phonological structure indicator (F_(1,41)_ = 0.413; *p* > 0.5), according to the group factor (students with dyslexia; control students). For instance, for the property indicator of phonological structure, we computed the average of this indicator of the words in each text, then the average obtained on the two texts of the same subject. Finally, we perform the ANOVA on both groups of subjects. The mean differences between the two groups for these three measures are not significant. Indeed, the words chosen and written by the students with dyslexia have a spelling consistency and a phonological structure comparable to the words chosen by the control students. The average indicator of grapheme-phoneme consistency is 72 (S.D. = 2.3) for students with dyslexia and 72.3 for control students (S.D. = 2.5). The average indicator of phoneme-grapheme consistency is 81.9 (S.D. = 1.9) for students with dyslexia and 82.2 for control students (S.D. = 1.9). The average indicator of phonological structure is 1.4 for students with dyslexia and control students (Dys. S.D. = 0.1; Control S.D. = 0.1).

#### 3.2.3. Frequency Indicator

Mean comparison tests (ANOVA) were performed on word frequency (F_(1,41)_ = 1.067; *p* > 0.1), according to the group factor (students with dyslexia; control students). Analyses show no significant results: students with dyslexia use words with similar frequency (6682; S.D. = 1191) as control students (6887; S.D. = 1045).

#### 3.2.4. Syntactic Properties

Mean comparison tests (ANOVA) were performed on the number of words belonging to the abbreviation (F_(1,41)_ = 0.389; *p* > 0.5), adjective (F_(1,41)_ = 0.830; *p* > 0.1), adverb (F_(1,41)_ = 0.000; *p* > 0.5), conjunction (F_(1,41)_ = 0.131; *p* > 0.5), determiner (F_(1,41)_ = 0.189; *p* > 0.5), noun (F_(1,41)_ = 0.070; *p* > 0.5), preposition (F_(1,41)_ = 0.621; *p* > 0.1), pronoun (F_(1,41)_ = 0.147; *p* > 0.5) and verb (F_(1,41)_ = 0.061; *p* > 0.5) categories, according to the group factor (students with dyslexia; control students). These numbers are related to the number of words in each text, so they are proportions. Consider, for example the category "adverb". We take all the adverbs of a text and relate this number to the total number of words of the text. We then take the average value obtained for the two texts produced by the same subject. Finally, we perform the ANOVA on the two groups of subjects. We observe an average of 72 adverbs per 1000 words in the texts of students with dyslexia and as many in the texts of control students. The ANOVA indicates that there is no significant difference. Students with dyslexia and control students use in a similar proportion abbreviated words (Dys: 0.001 (S.D. = 0.004); Control: 0.001 (S.D. = 0.003)), adjectives (Dys: 0.064 (S.D. = 0.026); Control: 0.060 (S.D. = 0.022)), conjunctions (Dys: 0.051 (S.D. = 0.021); Control: 0.053 (S.D. = 0.018)), determiners (Dys: 0.129 (S.D. = 0.028); Control: 0.126 (S.D. = 0.029)), nouns (Dys: 0.172 (S.D. = 0.030); Control: 0.174 (S.D. = 0.040)), preposition (Dys: 0.122 (S.D. = 0.023); Control: 0.117 (S.D. = 0.028)), pronouns (Dys: 0.124 (S.D. = 0.040); Control: 0.121 (S.D. = 0.040))and verbs (Dys: 0.172 (S.D. = 0.034); Control: 0.170 (S.D. = 0.036)).

Number of punctuation symbols was also analyzed (F_(1,41)_ = 5.842; *p* < 0.05) and the difference between the two groups is significant: control students have a higher proportion of punctuation symbols in their texts (0.106; S.D. = 0.022) than students with dyslexia (0.092; S.D. = 0.022).

### 3.3. Error Proportions

#### 3.3.1. Length Indicators

A mean comparison test (ANOVA) was performed on the proportion of words containing error(s) out of the total number of words comparing the group factor (students with dyslexia; control students) (F_(1,41)_ = 35.4539; *p* < 0.001).

The difference in average error rates between these two groups was highly significant. Analyses reveal that the dyslexic students’ written texts have a much higher average error rate (92 errors per 1000 words; 0.092; S.D. = 0.049) than the control students’ written texts (29 per 1000 words; 0.029; S.D. = 0.023), which represents a factor of 3.

#### 3.3.2. Length Indicators

Mean comparison tests (ANOVA) were performed on short (F_(1,41)_ = 29.036; *p* < 0.001), medium (F_(1,41)_ = 37.034; *p* < 0.001) and long (F_(1,41)_ = 17.052; *p* < 0.001) words containing error(s) as a proportion of the total number of words comparing the group factor (dyslexic; control students). Analyses reveal significant differences for the proportion of errors on short, medium, and long words between the two groups. The analyses reveal that the texts of students with dyslexia show:

- a proportion of errors on short words much higher (27 errors per 1000 words; proportion = 0.027; S.D. = 0.017) than control students (8 per 1000 words; proportion = 0.008; S.D. = 0.010);
- a proportion of errors on medium words much higher (30 errors per 1000 words; proportion = 0.030; S.D. = 0.020) than control students (7 per 1000 words; proportion = 0.007; S.D. = 0.007);
- a proportion of errors on short words much higher (36 errors per 1000 words; proportion = 0.036; S.D. = 0.028) than the control students (14 per 1000 words; proportion = 0.014; S.D. = 0.015).

#### 3.3.3. Frequency Indicator

Mean comparison test (MANOVA) was performed on the number of words containing error(s) according to frequency (as a proportion of the total number of words; F(2.77) = 14.469; *p* < 0.001) according to the group factor (students with dyslexia; control students) (Table 4).

Analyses revealed significant differences between students with dyslexia and control students for all observed variables: students with dyslexia make more errors than control students on words with a frequency of less than 10.8 (18 errors out of 1000 words vs. 8, for controls), on words with a frequency from 10.8 to 50.1 (21 errors out of 1000 words vs. 9, for controls), on words with a frequency from 50.1 to 567.6 (25 errors out of 1000 words vs. 8, for controls) and those with a frequency of more than 567.6 (28 errors out of 1000 words vs. 7, for controls).

#### 3.3.4. Graphemic and Phonetic Properties

Mean comparison tests (MANOVA, permitting to compare several values for one measure) were performed on the number of words containing an error according to the number of syllables, spelling consistency indicators, and phonological structure (as a proportion of the total number of words), according to the group factor (students with dyslexia; control students) (Table 5).

Analyses revealed significant differences between students with dyslexia and control students for all observed variables; students with dyslexia made:

- more errors than control students on words with one syllable (18 errors out of 1000 words vs. 10 for controls), with two syllables (33 errors out of 1000 words vs. 10 for controls), and those with more than two syllables (28 errors out of 1000 words vs. 13 for controls);
- more errors than control students on words with a grapheme-phoneme correspondence indicator lower than 61.3 (25 errors out of 1000 words against 8, for controls), between 61.3 and 72.6 (23 errors out of 1000 words against 7, for controls), between 72.6 and 79.5 (17 errors out of 1000 words against 9, for controls) and those higher than 79.5 (28 errors out of 1000 words against 9, for controls);
- more errors than control students on words with a phoneme-grapheme correspondence indicator lower than 79.5 (27 errors out of 1000 words against 8, for controls), between 79.5 and 86.9 (19 errors out of 1000 words against 9, for controls), between 86.9 and 96.2 (25 errors out of 1000 words against 8, for controls) and those higher than 96.2 (20 errors out of 1000 words against 8, for controls);
- more errors than control students on words with a phonological structure indicator of 0 (14 errors on 1000 words against 5, for controls), of 1 (29 errors on 1000 words against 10, for controls), higher than 1 (49 errors on 1000 words against 17, for controls).

#### 3.3.5. Syntactic Properties

Mean comparison tests (ANOVA) were performed on the proportions of adjectives (F_(1,41)_ = 14.632; *p* < 0.001), adverbs (F_(1,41)_ = 0.8257; *p* > 0.1), conjunctions (F_(1,41)_ = 0.263; *p* > 0.1), determiners (F_(1,41)_ = 12.789; *p* < 0.001), nouns (F_(1,41)_ = 20.24; *p* < 0.001), prepositions (F_(1,41)_ = 14.419; *p* < 0.001), pronouns (F_(1,41)_ = 3.848; *p* > 0.05), verbs (F_(1,41)_ = 40.631; *p* < 0.001) containing an error (relative to the total number of words in each text). There are no errors on abbreviations or punctuation. Analyses reveal significant differences regarding the proportion of errors on adjectives, determiners, nouns, propositions, and verbs by group. Results reveal that texts of students with dyslexia show:

- a higher proportion of errors on adjectives (13 errors per 1000 words; proportion = 0.013; S.D. = 0.011) than control students’ texts (4 per 1000 words; proportion = 0.004; S.D. = 0.007);
- a higher proportion of errors on determiners (3 errors per 1000 words; proportion = 0.003; S.D. = 0.004) than control students’ texts (0 per 1000 words; proportion = 0; S.D. = 0.001);
- a higher proportion of errors on nouns (27 errors per 1000 words; proportion = 0.027; S.D. = 0.011) than control students’ texts (8 per 1000 words; proportion = 0.008; S.D. = 0.011);
- a higher proportion of errors on prepositions (5 errors per 1000 words; proportion = 0.005; S.D. = 0.007) than control students’ texts (1 per 1000 words; proportion = 0.001; S.D. = 0.002);
- a higher proportion of errors on verbs (32 errors per 1000 words; proportion = 0.032; S.D. = 0.018) than control students’ texts (10 per 1000 words; proportion = 0.010; S.D. = 0.010).

Some categories do not show a significant difference, meaning that texts of students with dyslexia do not show significantly more errors on these types of words compared to control students: adverbs, conjunctions, and pronouns.

### 3.4. Errors Characteristics

The previous study revealed that the average proportion of errors in a text produced by a student with dyslexia is three times more important than that of a text produced by control students. We now turn to an analysis of the specific lexical properties to which students with dyslexia are the most sensitive. To do this, we divided the number of errors on a given property by the number of all errors in that text, in order to calculate “at constant error amount” whether distributions differ. We exploit exclusively the Corpus Word Error (the error corpus), which we divide into two sets: on one hand, the errors produced by students with dyslexia and on the other hand, the errors produced by control students. The latter is three times smaller than the former. We aim to discover which errors are over- or under-represented by comparing one set to the other, keeping in mind that there are three times more errors in the texts produced by students with dyslexia. Concretely, these are still proportions or mean values (cf. 6.2) but not calculated with respect to the number of words of the text, but to the number of errors found in that text.

#### 3.4.1. Length of Words Containing an Error

Mean comparison tests (ANOVA) were performed on the mean number of words containing error(s) syllables (F_(1,41)_ = 3.942; *p* = 0.05) and on short (F_(1,41)_ = 1.338; *p* > 0.1), medium (F_(1,41)_ = 0.965; *p* > 0.1) and long (F_(1,41)_ = 7.074; *p* < 0.05) words containing an error, according to the group factor (students with dyslexia; control students), in relation to the number of errors in the same text. Analyses show that the measures for which the differences between students with dyslexia and controls are significant are the number of syllables of words containing error(s) (Dys: mean = 2.0 (S.D. = 0.403); Control: mean = 2.2 (S.D. = 0.646)) and the number of long words containing errors (Dys: mean = 0.4 (S.D. = 0.215); Control: mean = 0.5 (S.D. = 0.281)). Mean differences are not significant for short (Dys: mean = 0.3 (S.D. = 0.178); Control: mean = 0.3 (S.D. = 0.250)) and medium (Dys: mean = 0.3 (S.D. = 0.153); Control: mean = 0.3 (S.D. = 0.266)) words. At constant error amount, students with dyslexia differed from the control students by making more errors on words with fewer syllables. Students with dyslexia make fewer long words containing an error than control students, that means that students with dyslexia make more errors on short and medium words.

#### 3.4.2. Frequency of Words Containing an Error

Mean comparison tests (ANOVA) were performed on the frequency of words containing error(s) (F_(1,41)_ = 4.012; *p* = 0.05), according to the group (students with dyslexia; control). Analyses show that frequency has a significant effect on errors. Students with dyslexia, using an error amount, differ from control students by making more errors on words with a higher frequency (mean frequency = 4169 (S.D. = 3560); mean frequency = 2867 (S.D. = 4058)): students with dyslexia make errors on words 50% more frequent than control students.

#### 3.4.3. Graphemic and Phonological Properties of Words Containing Error(s)

Mean comparison tests (ANOVA) were performed on spelling consistency indicators (grapheme-phoneme consistency (F_(1,41)_ = 0.023; *p* > 0.5); phoneme-grapheme consistency (F_(1,41)_ = 0.225; *p* > 0.5) and phonological structure (F_(1,41)_ = 0.930; *p* > 0.1), according to the group factor (students with dyslexia; control students). For instance, for the property “the phonological structure”, we compute the average of the phonological structure indicator of the erroneous target words of each text, then the average obtained on the two texts produced by the same subject. Finally, we perform the ANOVA on both groups of subjects. Analyses show no significant differences between the two groups for these three measures. Indeed, the average indicator of grapheme-phoneme consistency of words containing error(s) is 68.2 (S.D. = 9.5) for students with dyslexia and 69 for control students (S.D. = 12.1). The average indicator of phoneme-grapheme consistency of words containing errors(s) is 85.5 (S.D. = 5.3) for students with dyslexia and 84.5 for control students (S.D. = 8.2). The average indicator of the phonological structure of words containing error(s) is 1.6 (S.D. = 0.3) for students with dyslexia and 1.8 (S.D. = 0.8) for control students.

#### 3.4.4. Syntactic Properties

Mean comparison tests (ANOVA) were performed on the proportions of adjectives (F_(1,41)_ = 0.034; *p* > 0.5), adverbs (F_(1,41)_ = 1.127; *p* > 0.1), conjunctions (F_(1,41)_ = 1.545; *p* > 0.1), determiners (F_(1,41)_ = 7.222; *p* < 0.05), nouns (F_(1,41)_ = 0.034; *p* > 0.5), prepositions (F_(1,41)_ = 4.551; *p* < 0.05), pronouns (F_(1,41)_ = 0.626; *p* > 0.1) and verbs (F_(1,41)_ = 0.062; *p* > 0.5) containing an error (related to the number of errors), according to the group factor (students with dyslexia; control students). Analyses show that the only measures for which there are significant differences between students with dyslexia and controls are the number of words containing an error belonging to the grammatical category of determiners and prepositions. The error amount for students with dyslexia differs from control students by making more errors on determiners and prepositions. Results reveal that texts of students with dyslexia show:

- a higher proportion of errors on determiners (34 errors per 1000 words; proportion = 0.034; S.D. = 0.048) than control students’ texts (9 per 1000 words; proportion = 0.009; S.D. = 0.001);
- a higher proportion of errors on prepositions (5 errors per 1000 words; proportion = 0.005; S.D. = 0.007) than control students’ texts (1 per 1000 words; proportion = 0.001; S.D. = 0.033);

Some categories do not show a significant difference, meaning that texts of students with dyslexia do not show significantly more errors on these types of words compared to control students, when we restrict analyses to the errors: adjectives (Dys: mean = 0.1 (S.D. = 0.1); Control: mean = 0.1 (S.D. = 0.2)), adverbs (Dys: mean = 0.037 (S.D. = 0.057); Control: mean = 0.061 (S.D. = 0.153)), conjunctions (Dys: mean = 0.008 (S.D. = 0.018); Control: mean = 0.038 (S.D. = 0.168)), nouns (Dys: mean = 0.3 (S.D. = 0.151); Control: mean = 0.3 (S.D. = 0.2799)), verbs (Dys: mean = 0.4 (S.D. = 0.136); Control: mean = 0.3 (S.D. = 0.223)) and pronouns (Dys: mean = 0.07 (S.D. = 0.102); Control: mean = 0.05 (S.D. = 0.082)).

## 4. Discussion

As mentioned in the literature review, students with dyslexia have difficulties with written text production, whether it be with syntax, text organization, revision, and of course spelling [1,2,4,26,43,44,45,46,47,48,49,50,51,52,53]. The initial objective of this current study was to complete the existing work on spelling and better identify the spelling difficulties to improve the remediation and to support students with dyslexia more efficiently. We wanted to precise the spelling difficulties of students with dyslexia. If it exists, a lot of studies about children with dyslexia in the psychology and neuropsychology domains, search literature revealed few studies about adults or students and moreover in a psycholinguistic point of view. It seems indeed obvious, according to the international literature, that dyslexic students make more spelling errors, but what kind of errors? We wanted to go further and to complete the existing studies by observing, in particular, the effect of the type of word, and according to our knowledge of the domain, no previous study has investigated spelling difficulties in this perspective. For instance, does a word with a weak spelling consistency generate more errors? And what about a word with a low frequency? Or a long word? With respect to the previous studies, we hypothesized that students with dyslexia: (1) use less complex words than control students, such as shorter words, words with regular spelling or frequent words (Hypothesis 1, H-1); (2) overall, make more errors than control students, and this is true for all types of words (Hypothesis 2, H-2); and (3) are more sensitive to certain linguistic properties and therefore make more errors on certain types of words, compared to control students (Hypothesis 3, H-3). To examine these hypotheses, we ran several analyses on four types of measures: length (number of syllables and word length in letters); graphemic and phonetic (the phoneme/grapheme and grapheme/phoneme consistency indicators and the phonological structure); frequency (frequency of lemmas); syntactic (grammatical class of words).

To check our first hypothesis, we ran some analyses on the previously mentioned measures on all the words used by students with dyslexia and control students as a proportion of the total number of words in the written text. Previous studies conclude that students with dyslexia do not make the same lexical choices during a written task as control students do [1,26,50]. In this sense, we have hypothesized that students with dyslexia of the present study would experience linguistic insecurity feeling and would tend to use shorter words (in terms of the number of syllables and letters), words with regular spelling (so with bigger grapheme-phoneme and phoneme-grapheme consistency) and more frequent words. Several studies reported that students with dyslexia do not produce polysyllabic words as much as control students do [1,26,50] and have a smaller vocabulary [1,50,58]. Moreover, their difficulties with writing, and more specifically with spelling [26,28,47,48], push them to avoid the use of certain words, for which they are not sure about the spelling [1,26]. In contrast to these earlier findings, our analyses (Section 3.2) did not confirm these differences between students with dyslexia and control students. Unexpectedly, students with dyslexia, indeed, produce the same type of words as control students: they use words with the same length, the same frequency, and the same spelling consistency. Indeed, results reveal no significant difference between the length (Section 3.2.1) the spelling and phonological structure (Section 3.2.2), consistency and frequency (Section 3.2.3) of students with dyslexia and control students (Actually, only one significant difference has been observed and concerns the use of punctuation. Although our experimental plan involved a number of students being fairly acceptable with respect to what is standard in the field, we acknowledge that those numerous and unexpected non-significant differences should be confirmed by an additional analysis of statistical power). We can conclude that students with dyslexia of this sample use and write the same type of words in their written texts and in the same proportion. Hypothesis 1 was not verified, and this was an unexpected outcome. We thought, indeed, that aware of their disorder and difficulties with spelling, students with dyslexia would censor themselves. We expected them to make different lexical choices than the control students, but they did not. There are no over or under-represented words categories that may reflect inhibition or overuse in students with dyslexia. This result shows also that spoken language capacities are rather well preserved in students with dyslexia [79,80,81].

To check our second hypothesis, we ran some analyses on the previously announced measures on all the wrong words used by students with dyslexia and control students as a proportion of the total number of words in the written text. Prior studies reveal that students with dyslexia realize more spelling, syntactic [26,28,47,48] and morphosyntactic errors [1,56,57]. The results of this current study are in line with those of previous studies. Indeed, we can see that students with dyslexia realize more errors on long, medium, and short words than control students (Section 3.3.2 and Section 3.3.4). Regarding frequency (Section 3.3.3), results show that, again, regardless of word frequency, students with dyslexia make more errors than controls. Results bring additional surprising information: control students made almost the same proportion of errors on both frequent (Q4) and rare (Q1) words, whereas students with dyslexia had a rather surprising error distribution: indeed, the proportion of words containing an error of students with dyslexia with high frequency (Q4) is higher than the proportion of words containing an error of lower frequency (Q3, Q2, and Q1). Concerning spelling consistency, we can see that students with dyslexia make more errors than control students on all types of words, whether they have a high or low consistency indicator. For phonological structure, students with dyslexia again make more errors than control students, regardless of the level of phonological structure. Finally, concerning morphological properties, we found that students with dyslexia made more errors on verbs, nouns, adjectives, and determiners; they make mistakes on prepositions too whereas control students did not at all (Section 3.3.5). For adverbs, conjunctions, and pronouns, there are no significant differences between the two groups. Again, the distribution of errors according to the grammatical class of words are quasi the same between students with dyslexia and control students: both made more errors on first verbs, then nouns and adjectives. Another important finding was observed for prepositions and determiners: control students do not make mistakes on these types of words, whereas students with dyslexia do. This is a surprising result as they are rather frequent and short words. Overall, Hypotheses 2- is confirmed: we observe a very significantly higher number of errors in the texts produced by students with dyslexia than in the texts produced by controls, which is in line with previous studies [1,43,45,47,51,56,57]. With a few exceptions, this occurs in all categories of words: frequent or infrequent, short or long, invariant grammatical words, or highly inflected words (verbs, nouns, adjectives). Knowing that these categories of words are identically distributed in the texts produced by both, we can conclude that errors do not seem to depend on the lexical, grammatical, or phonological properties of the target word. The errors seem to “fall” indiscriminately on all word categories by a factor of 3. Moreover, we would expect that long words would be more impacted and words with fewer syllables and fewer letters (most often invariable, tool words) less impacted but, this is not the case. To conclude, the experimental response is quite clear: students with dyslexia make significantly more errors than controls and this seems to affect all word categories indiscriminately.

To check our third hypothesis, we ran analyses on the measures announced above on all the wrong words used by students with dyslexia and control students as a proportion of the total number of wrong words in the written text. Concerning the length indicators, two measures, “number of syllables of words containing an error “ and “number of long words containing an error “ show significant differences between students with dyslexia and controls: students with dyslexia differ from controls by producing more errors on short words (in number of syllables) and less errors on long words (in number of letters) than control students (Section 3.4.1); that means that students with dyslexia make more errors on short and medium words (in terms of letters). These results can be surprising; we expected the opposite. What poses more spelling difficulties for students with dyslexia would be the smallest words, not the long ones, which is the case for the control students. Concerning frequency, results are also surprising: contrary to the errors of the control students, the errors of the students with dyslexia are observed more on frequent words. Concerning graphemic and phonetic properties, we expected that low spelling consistency and high phonological structure would be reasons for errors of students with dyslexia, more than for control students. However, results show that students with dyslexia, on equal measures, do not make more errors than control students when the words have a high spelling consistency indicator and a high phonological structure indicator (Section 3.4.3). Concerning syntactic properties, here again, the results are unexpected. We expected differences in the proportions of words containing an error between the two groups, especially for nouns, verbs, and adjectives, which are, in French, subject to important inflectional variation. However, analyses show that, for these categories, students with dyslexia do not differ from control students: they do not produce more errors than the control students on these types of words. In contrast, when we compare the proportion of words containing an error on determiners and prepositions between the two groups, results show significant differences: on equal measures, students with dyslexia make more errors on these types of small words than control students. Hypotheses 3- is also partially confirmed: students with dyslexia are indeed sensitive to certain types of words, but not those we expected. Errors of students with dyslexia are over-represented where they are not expected: on short and frequent words, on determiners and prepositions. They are not over-represented in expected categories, such as: long, complex (spelling consistency and phonological structure), rare, and highly inflected words. It is therefore not lexical or grammatical complexity that seems to be a problem for students with dyslexia, but properties present in particular on grammatical words, even if these seem “simple” and usual. If these findings can be contrary to some previous studies which have suggested that students with dyslexia made more mistakes on long and rare words for instance [26, 53], they confirm few recent results. Indeed, in other studies [1,56,57], we can find that English students with dyslexia have difficulties with determiner and prepositions, implying two problems: omission and confusion. Recent literature confirms our results: students with dyslexia can have some difficulties with short and frequent words [82] and the ten most words containing an error are short and frequent (très, peut, à, après, ils, ont, c’est, ce, au, est). Cidrim & Madeiro (2017) [45] confirm that there is a lack of studies on short and frequent words. To conclude, it is more the spelling process of the invariant words which is concerned and is a problem for students with dyslexia.

This current study permits to more precisely understand spelling difficulties of students with dyslexia, even if certain results can be surprising. How can we explain these results?

Firstly, we have to remember that we are studying the texts of successful students with dyslexia who are in higher education. Students with dyslexia develop compensations that allow them to manage the constraints of their studies, which strongly interfere with their disorder, whereas they are intensively exposed in writing [80]. These compensations do not eliminate the disorders and the difficulties, but they are effective enough for students with dyslexia to progress in higher education [80]. Studies about these compensations are relatively few compared to ones on deficits, but we can understand this perhaps given that there are several types of compensations [83]: neurobiological, cognitive, and socio-emotional. If there are not many studies and consensus about compensations, it seems that compensations appear like a protection factor. Among these protection factors, some recent studies mention morphology abilities that permit teenagers with dyslexia [84] and students [85] to compensate for their reading difficulties. An additional study mentions vocabulary skills that can compensate for reading and phonological structure difficulties [83]. We can also ask if these morphological and vocabulary skills can have a positive impact on writing, as well as on reading. If they do, that can explain why students with dyslexia in our study used the same type of words as control students and why students with dyslexia do not make more errors than control students on certain types of words, such as nouns, verbs, or adjectives. For these lexical words, students with dyslexia can rely on their morphological abilities. The same possible explanations can be given for results about frequency: their vocabulary skills could help them, and they also use words with low and high frequency, just as the control students do. These compensations could, however, have their own limits: short and frequent words, and more precisely prepositions and determiners. We also suggest that these types of words imply three difficulties for students with dyslexia: (1) for these words, it seems to be less obvious to make use of morphological abilities; (2) short and frequent words can be homophones; previous studies reported that students with dyslexia have considerable difficulties with homophones [47]; (3) the difficulties in handling these small words can be due to the fact that they are more laborious to memorize than lexical meaningful words: there is less anchoring in semantic memory. The connectionist PDP (Parallel Distributed Processing) model [86] emphasizes the fact that reading networks are composed of orthographic, phonological, and semantic identification skills that are connected to each other and work in synergy [86]. These small meaningless words—prepositions and determiners—are totally dependent on their co-textual environment: they are dependent from lexical units.

Secondly, another possible explanation for this might be that spelling difficulties could be linked to a lexical and spelling deficit. Indeed, some recent studies mention that spelling difficulties of students with dyslexia are not exclusively due to phonological process but also to a deficit that affects lexical and orthographic processing [43,45], which can explain their specific spelling difficulties with short words.

Thirdly, these results may be explained by the relationship between dyslexia and attention deficit hyperactivity disorder (ADHD); there exists indeed strong comorbidity between dyslexia and ADHD [87]. We know that students with dyslexia in our study do not have an attention deficit hyperactivity disorder, but previous analyses reveal that they have different attentional behavior from the control students [4]: attentional tests indicate that students with dyslexia are significantly more impulsive and distractable than control students. Moreover, they present a lack of attention during long and monotonous tasks. Without being pathological, this attentional fragility can also explain the fact that students with dyslexia have an over-representation of errors on small words. It is indeed one of the frequent errors of people with ADHD [85,86,87,88,89].

## 5. Conclusions

### 5.1. Perspectives

This present study allows for three main conclusions. The first major finding is that students with dyslexia, in their written texts, use the same types of words as control students. This is an unexpected result and students with dyslexia do not seem to experience linguistic insecurity, or at least, if they do, it is not visible in the analyses of their written productions. They do know that they have difficulties with spelling, but they do not inhibit themselves by using specific words. The second major outcome is that students with dyslexia make almost three times more errors than control students and in all word categories. This is also a surprising result, in the sense that we could have expected that some word categories would be less impacted than others. The fact that they make a lot of errors and everywhere is consistent with the first point previously stated: not having a target identified as “more problematic”, they cannot know, for instance, which target to avoid. Finally, the third major conclusion of this study is that, on the one hand, students with dyslexia are not particularly sensitive to complex words (from a lexical and grammatical perspective). On the other hand, they are widely more sensitive than control students to short and frequent words, such as prepositions and determiners.

### 5.2. Limits of the Present Study

That being said, we also want to point out the limitations of this current study. The major limitation of this study is that we use the category of dyslexia without being able to distinguish the underlying deficits. There are relatively few studies that have characterized developmental dyslexia in adults, and it seems very difficult to identify precisely the type of deficits involved in adults [4]. Habib [90] describe three profiles of dyslexia for children, linked to several types of disorders: linguistic, attentional, or motor, which may suggest potentially different mechanisms of origin, and with specific clinical impacts. Moreover, given the lack of studies in the field of dyslexia in adulthood, we are forced to build upon work dyslexia during childhood. Finally, we used Manulex-Infra to have spelling consistency indicators, based on school textbooks. But we attempted to remedy this limitation by re-using the algorithm set up for this consistency calculation on words not referenced by Manulex-Infra.

Additional studies could bring more precise responses to many questions. Firstly, a more qualitative study about these short words containing an error in order to have more information about these errors on short words. We know that homophones are difficult to handle for a person with dyslexia [1,27,47,48]. In this sense, we are for now conducting analyses on short words, and more specifically on homophones, to see if students with dyslexia of our project make more homophonic substitution errors than their peers and what can explain these differences in the categories between the two groups. Moreover, as mentioned before, we want to test the effect of different attentional behavior of the students with dyslexia compared to the control students [4] on the treatment of these short words.

Secondly, a study on the impact of frequency and consistency on motor execution, as observed in chronometric measures of writing durations. Indeed, previous works on Spanish children with dyslexia show a significant impact of consistency and frequency on sentence dynamics (on-line processes) [91]. It would also be interesting to observe the location of these words in the clause and the associated pause phenomena.

## Figures and Tables

**Table 1:** Description of participants in the psycholinguistic task.

	Students with Dyslexia	Control Students
Mean age	21.7	21.8
Range	18.1–28.5	18.1–28.9
Total number of subjects	21	22
According to gender	9 women/12 men	10 women/12 men

**Table 2:** Length indicators for written texts according to text type and group.

	Expository Texts		Narrative Texts	
	Students with Dyslexia	Control Students	Students with Dyslexia	Control Students
Mean number of words per text	198.2 (101)	181.1 (139)	207 (131)	181 (112)
Mean number of clauses per text	25.8 (12.7)	25 (17)	30.7 (20)	27 (16)
Mean number of TU per text	12 (5)	12 (9.4)	14.7 (9.9)	13.8 (8.2)
Mean number of clauses per TU	2.1 (0.5)	2.2 (0.4)	2.1 (0.3)	2.04 (0.5)
Mean duration of production (in minutes)	13.85 (8.60)	11.02 (8.16)	11.77 (7.60)	9.01 (4.94)

**Table 3:** Length indicators for written texts according to text type and group.

	Expository Texts		Narrative Texts			
	Students with Dyslexia	Control Students	Total	Students with Dyslexia	Control Students	Total
Number of words	4161	4017	8178	4425	4099	8524
Number of errors	502	94	596	461	120	581

**Table 4:** Average number of errors (w.r.t. text length) according to 4 levels (quartiles Q1, Q2, Q3 and Q4) of frequency.

Group	N	Q1	Q2	Q3	Q4	MANOVA	
Quartiles		<10.8	10.8–50.1	50.1–567.6	>567.6		
Dys.	42	0.018	0.021	0.025	0.028	F(2.77) = 14.469; *p* < 0.001	S
Control	44	0.008	0.009	0.008	0.007		

**Table 5:** Average number of errors (with respect to (w.r.t.) text length) according to 4 levels of each indicator of graphemic/phonetic.

Group	N	Q1	Q2	Q3	Q4	MANOVA	
Number of syllables							
Levels		1	2	>2			
Dys.	42	0.018	0.033	0.028		F(3.78) = 4.0857; *p* < 0.01	S
Control	44	0.010	0.010	0.013			
Indicator of grapheme-phoneme consistency							
Levels		<61.3	61.3–72.6	72.6–79.5	>79.5		
Dys.	42	0.025	0.023	0.017	0.028	F(2.77) = 12.829; *p* < 0.001	S
Control	44	0.008	0.007	0.009	0.009		
Indicator of phoneme-grapheme consistency							
Levels		<79.5	79.5–86.9	86.9–96.2	>96.2		
Dys.	42	0.027	0.019	0.025	0.020	F(2.77) = 12.370; *p* < 0.001	S
Control	44	0.008	0.009	0.008	0.008		
Indicator of phonological structure							
Levels		0	1	>1			
Dys.	42	0.014	0.029	0.049		F(3.78) = 3.3536; *p* < 0.05	S
Control	44	0.005	0.010	0.017			

## Data Availability

Data are not publicly available. Please contact the authors for any information.
